# Predicting hospital cost in CKD patients through blood chemistry values

**DOI:** 10.1186/1471-2369-12-65

**Published:** 2011-12-01

**Authors:** Russell W Bessette, Randy L Carter

**Affiliations:** 1 University of Louisville, Abell Administration Center, 323 East Chestnut St. Louisville, Kentucky 40202, USA; 2 State University of New York at Buffalo, School of Public Health, Department of Biostatistics and Population Observatory, Farber Hall, Main St, Buffalo, New York 14214, USA

## Abstract

**Background:**

Controversy exists in predicting costly hospitalization in patients with chronic kidney disease and co-morbid conditions. We therefore tested associations between serum chemistry values and the occurrence of in-patient hospital costs over a thirteen month study period. Secondarily, we derived a linear combination of variables to estimate probability of such occurrences in any patient.

**Method:**

We calculated parsimonious values for select variables associated with in-patient hospitalization and compared sensitivity and specificity of these models to ordinal staging of renal disease.

Data from 1104 de-identified patients which included 18 blood chemistry observations along with complete claims data for all medical expenses.

We employed multivariable logistic regression for serum chemistry values significantly associated with in-patient hospital costs exceeding $3,000 in any single month and contrasted those results to other models by ROC area curves.

**Results:**

The linear combination of weighted Z scores for parathyroid hormone, phosphorus, and albumin correlated with in-patient hospital care at p < 0.005. ROC curves derived from weighted variables of age, eGFR, hemoglobin, albumin, creatinine, and alanine aminotransferase demonstrated significance over models based on non-weighted Z scores for those same variables or CKD stage alone. In contrast, the linear combination of weighted PTH, PO4 and albumin demonstrated better prediction, but not significance over non-weighted Z scores for PTH alone.

**Conclusion:**

Further study is justified to explore indices that predict costly hospitalization. Such metrics could assist Accountable Care Organizations in evaluating risk adjusted compensation for providers.

## Background

Patients with chronic kidney disease (CKD) are at risk for complications requiring costly hospital care. However predicting which patients are at risk for high cost hospitalization has been a difficult endeavor. Cardiovascular disease (CVD) is a co-morbidity that drives many costs [1-3]. In addition, CKD patients often display dysfunction of the endocrine system that distorts the balance between calcium, phosphate and parathyroid hormone leading to calcification of the arterial tree and aggravation of hypertension and CVD along with costly hospitalization [4-6].

Some investigators have suggested that serum PTH levels can predict cardiovascular events [7-9]. Numerous animal studies have confirmed these observations for elevated PTH levels [10-13]. Further, it is suggested that high levels of PTH contribute to hyperlipedemia and impaired glucose tolerance [14-17].

Disorders in mineral metabolism have become a focal point for predicting costly hospitalization. Covic et al in a comprehensive review of the literature reported a significant rise in all-cause mortality associated with serum mineral disturbances [18]. Their review suggested that abnormal plasma levels of phosphorus, followed by calcium and parathyroid hormone increased mortality. However, as cautioned by the authors, the most articles reported on patients with end stage renal disease (ESRD). In a subsequent study, Bhuriya et al analyzed PTH levels in patients with CVD and stage 3 and 4 CKD [19]. Employing multivariable logistic regression analysis, they reported on the association of age, hemoglobin level, eGFR, plasma PTH, phosphorus, and calcium levels with CVD events. Their analysis demonstrated that PTH levels greater than 70 pg/ml increased the risk for CVD significantly. However, they found no relationship with levels of serum phosphorus or calcium.

On the other hand, other investigators have demonstrated that elevated alkaline phosphatase predicted mortality and hospitalization in hemodialysis patients independent of calcium, phosphorus, and PTH levels [20].

Serum albumin associated with calcium binding is another factor linked with increased morbidity in CKD patients. Since low serum albumin is frequently seen with protein energy malnutrition, it has been suggested as a predictor for increased mortality in CKD patients [21-23].

In summary, chronic kidney disease is clearly associated with multiple organ dysfunctions that impact cost, diminish health and work productivity [24-29]. However as seen in the literature, there is disagreement as to which blood chemistry values reliably predict illness complexity and high cost healthcare. Since CKD afflicts up to 20 million Americans and accounts for annual dialysis costs of $121,000 per patient with ESRD, the ability to predict high-cost care in chronic disease is vital for future Accountable Care Organizations. In this study, we investigated if measurement of blood chemistry values would add additional information over ordinal staging of renal disease and therefore improve the management of CKD patients.

We analyzed the association of each blood chemistry variable on the probability of hospitalization, and did so under the assumption that the influence of each variable is through its impact on a linear predictor. The result provides an association of each variable to the probability for an adverse outcome.

## Objective

The objective of this study was to investigate the relationship between select serum chemistry values and the occurrence of in-patient hospital payments exceeding $3,000 in any single month for a range of CKD patients. Next, we compared those results to other predictive models based on non-weighted Z scores of the same serum values or to ordinal stages of CKD in the same patients.

## Method

### Samples Analyzed

Our data set included 1104 de-identified patients from the kidney disease registry of a local managed care organization (MCO), who had received treatment from November 2007 through November 2008. This data was obtained after review of the study protocol by the university's school of medicine Institutional Review Board (IRB) and permission from the regional MCO.

Since our goal was to study the relationship between a select series of blood chemistry values and high hospital cost in CKD patients, we employed the definition of CKD as two or more recordings of eGFR at or below 60 ml/min over a three month period. In the dataset supplied to us, CKD was confirmed in 888 patients. The remaining 216 excluded patients had no eGFR values recorded at any time over the 13 month study period. In addition within this excluded group, 212 patients had one or more serum glucose recordings, and 139 had one or more abnormal serum creatinine values with no other blood tests recorded at any time. These excluded patients appeared on the renal registry because their primary-care physician assigned a diagnostic code (ICD-9) indicating acute or chronic renal disease or a family history of CKD with diabetes.

### Variable Definitions

A total of eighteen blood tests were requested from the MCO for analysis in this study by a consulting group of university nephrologists. The test choices were made based on each variable's perceived importance in monitoring the health of CKD patients. The 18 blood tests were: serum urate, phosphorus (PO4), parathyroid hormone (PTH), glucose, glycolated hemoglobin (HbA1c), hemoglobin (HGB), bicarbonate, albumin, creatinine, urea nitrogen (BUN), potassium, calcium, sodium, alkaline phosphatase, alanine aminotransferase (ALT), bilirubin, leukocytes, and eGFR (by MDRD4). The data set also included the complete financial profile for all medical claims that were paid for these patients over the same time period. These costs were linked to lab records using SQL queries written to join lab and claims data by unique patient identifiers within each dataset and allowed reimbursements to be studied.

Since tests ordered by physicians showed marked variation in selection and repetition, we sorted the remaining pool of 888 patients into two data sets for two separate sets of modeling analyses based on the following criterion: (1), 267 patients with no missing observations for serum parathyroid hormone (PTH) in order to focus on mineral metabolism disorders; and (2) 792 patients with no missing observations for serum creatinine in order to focus on serum values associated with renal function. Several models with various sets of explanatory variables were fitted using each of these data sets. Each model was fitted to the data for the subset of all patients in the respective data sets with non-missing values for every variable in the model.

The blood tests for all patients were performed by the same laboratory. Thus, the units of measurement and normal range for each test were common to all observations. Summary measures over the 13-month observation period were calculated for each lab test by averaging the tests results over all times of observation. Except for HbA1c and eGFR, the mid-point of the normal range for each test was taken as the mean, and the range divided by four as the standard deviation for a non-diseased normal population. Each lab test was standardized using this mean and standard deviation to obtain a Z-score for each variable for each patient.

Costs were totaled within each month and used, along with the codes for service provider type (e.g. hospital, surgery, internal medicine, nephrology, family medicine, pharmacy, etc) to define cost allocation. The frequency distribution for all hospital reimbursements displayed a bimodal curve with $3000 as the best dividing point between the two modes. We therefore selected this value as a cutoff point. This decision was further justified by the fact that total payments which exceeded $3000 were primarily for in-patient hospital care. That variable was defined as outcome 1. We chose to name this outcome as "High-Cost Hospitalization" or HCH to allow for the possibility that hospital care could be associated with reimbursements less than $3000. Other patients not meeting this criterion were assigned an outcome value of 0, or non-HCH.

### Study Outline

The primary purpose of our study was to test the associations between a CKD patient's serum chemistry values and the occurrence of HCH in any single month over the thirteen-month study period. Secondarily, we derived a linear combination of blood tests to estimate the probability of HCH for any given patient. Next, we tested the association of a patient's CKD stage to the occurrence of HCH under the same criterion. Next the sensitivity and specificity for predicting HCH was calculated for a sequence of cut-points on each linear predictor scale by comparing predicted values of HCH to the occurrence of true positive and true negative HCH. Finally, the predictive models were compared through calculation of areas under receiver operating characteristic (ROC) curves.

### Statistical Methods

As discussed previously, some investigators conclude that measurement of PTH, phosphorus, calcium, alkaline phosphatase, albumin and eGFR predict illness severity and hospitalization in renal patients. To test for this association, we modeled the probability of HCH as a multivariable logistic function of average age, eGFR, and the Z-scores calculated from the average measures of PTH, phosphorus, bicarbonate, albumin, potassium, calcium, sodium, alkaline phosphatase and eGFR, over the 13 month period. A backward selection model building strategy was employed to derive a parsimonious model containing only significant predictors. At each step, the explanatory variable with the highest p-value greater than 0.10 was deleted. If its deletion resulted in another variable that had been significant (p < 0.10) previously becoming non-significant, then the deleted variable was added back into the model and the variable with the next largest p-value greater than 0.10 was deleted. These steps were repeated until only significant variables (p < 0.10) remained in the model.

These analyses produced a regression table with an estimated constant and regression coefficients for each explanatory variable in the final model, along with calculated p-values. The Hosmer-Lemeshow Goodness-of-Fit test was calculated to test for a lack of fit of the final model. Probability curves were created relating the linear predictor (*i.e.*, the weighted sum of predictor variables with weights that are the estimated coefficients from the logistic regression) to the probability of HCH.

The initial data set focused on analyses of mineral metabolism and contained 267 patients; the second analysis focused on 792 patients and used other available blood chemistry values. Employing the data set with 792 patients, the multivariable logistic regression model building strategy described above was employed to derive a parsimonious model containing the significant predictors for HCH from among the following variables: age and Z-scores for blood glucose, hemoglobin, bicarbonate, albumin, creatinine, urea nitrogen, potassium, calcium, sodium, alkaline phosphatase, ALT, and white blood cell count (leukocytes). As above, the goodness of fit of the final model was tested using the Hosmer-Lemeshow test.

For the logistic regressions described above, the number of observations used to fit each model was the number with non-missing values of all variables in the model. All computations were done using the Minitab package of statistical software.

We calculated the sensitivity and specificity for each model based on a series of cut-points on the linear predictor scale for the final multivariable logistic regression models. We then compared resulting predicted values to the occurrence of true positive and true false values for HCH and calculated sensitivity and specificity for each cut-point. The same calculations were made for cut-points on the linear predictor obtained from the CKD stage model for predicting HCH. Similarly, calculations were made for a series of cut-points on the linear predictors defined by the sum of non-weighted Z scores in both the mineral metabolism and renal models for the occurrence of HCH. Lastly, ROC curves were calculated for each model along with an area under each respective curve in order to compare models for accuracy in predicting HCH. ROC curves and areas under the curves were calculated using the software application by Eng J. ROC analysis: web-based calculator for ROC curves. Baltimore: Johns Hopkins University [updated 2006 May 17.] Available from: http://www.jrocfit.org.

## Results

For the total pool of CKD patients in this study, analysis of the claims data revealed that 435 patients had at least one HCH (i.e., HCH = 1) month. The remaining 453 patients had no HCH during the 13 study-months (HCH = 0).

The average annual payment per patient for the group designated non-HCH (outcome 0) was $3,167 with a range of $264 to $17,197. The average monthly payment per patient in this group was $313.

In contrast, the HCH (outcome 1) group had average annual payments of $35,892 with a range of $4,276 to $314,533. Their average monthly per patient payment was $3,136.

**Figure 1:**
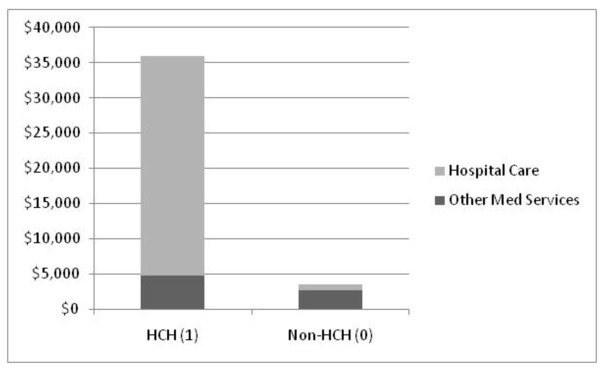
**Comparison of High Cost Hospital (HCH) group to non-High Cost Hospital (non-HCH) group**. Stacked histogram comparing average annual total per patient healthcare payments in the HCH and non-HCH groups. Black equals payments for physicians and other medical services; gray equals payments for hospital services only.

Figure 1 is a stacked histogram demonstrating the average yearly payments per patient. Payments for hospital only services are shown in light gray, and payments for other medical services shown in black for both the HCH and non-HCH groups. In the HCH group, payments for hospital only services averaged $31,242 per patient with a range of $3,068 to $307,906. Other medical services for those same patients had average annual payments per patient of $4,671 with a range of $55 to $25,153.

On the other hand in the non-HCH (outcome 0) group, payments for hospital only services averaged $830 per patient with a range of $0 to $5,865. For other medical services, that average total payment per patient was $2,652 with a range of $264 to $16,572.

For the 267 patients with repeated PTH and serum phosphate testing, logistic regression analysis demonstrated a significant association between increasing PTH levels and HCH at p < 0.005. The Hosmer-Lemeshow Goodness-of-Fit test p-value was calculated at 0.06 with 66.5% concordant pairs between the response variable and the predicted probabilities.

For those variables associated in the literature with mineral metabolism disorders (age, PTH, phosphorus, bicarbonate, albumin, potassium, calcium, sodium, alkaline phosphatase, eGFR) their overall p-value for correlation with HCH was significant at p < 0.005, nonetheless, a number of variables had p-values that were not significant. After a step-wise elimination of the least significant variable, the regression calculation for the most parsimonious model demonstrated that PTH, phosphorus and albumin had significance at p < 0.005 with a Chi-Square Goodness of Fit test that was not significant (p = 0.83). In addition, there was an association of 74.3% concordant pairs between the response variables and predicted probabilities.

Using the calculated regression coefficients for the linear predictor's constant and PTH, phosphate, and albumin coefficients, we calculated a probability curve for HCH as a function of the linear predictor, using the following formula for probability of HCH given e^lp^/(1+e^lp^), where

lp = -1.21+0.03*PTH z-score + 0.36*PO4 z-score -0.54 * albumin z-score.

**Figure 2:**
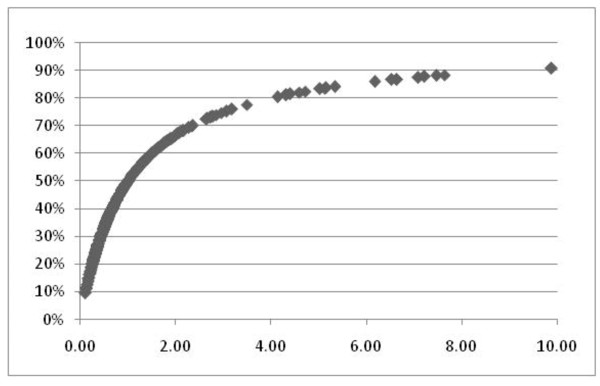
**Probability curve for patients with hospital care costs exceeding $3,000 monthly**. Probability of Hospital Care exceeding $3,000 in any single month versus the linear predictor (lp) of the logit calculation for average serum levels of PTH, phosphate and albumin.

By calculating e^lp^/(1+e^lp^) for each patient and plotting versus the linear predictor (lp) we produced the curve shown in Figure 2.

The probability for HCH increased sharply to 50% as the linear predictor for serum PTH, phosphorous and albumin increased from 0.0 to 1.0. With an increase of the linear predictor to 2.0, the probability for HCH rose to 65%. As the linear predictor increased to 4.0, the probability for HCH reached 80%. And as the linear predictor doubled from 4.0 to 8.0, the probability of HCH increased to 90%.

In order to tabulate the impact of individual variables on the outcome of HCH, we calculated individual probability curves for PTH, phosphorus and albumin. By holding each of the non-selected variables at Z-score = 0, we recalculated logistic regression values and subsequent probability values. For Z-scores of PTH at 20, 40 and 70, the probability of HCH was 34%, 50% and 72% respectfully. For Z-scores of phosphorus at 2, 4, 6, the probability of HCH was 36%, 55%, and 70% respectively. For Z-scores of albumin at -2.0, -3.0 and -4.0, the probability of HCH was 42%, 55%, and 69% respectively.

Since the reference range for normal can vary in different laboratories, practicing clinicians can calculate the Z-scores for their patient's test values and substitute those values within the above formulas in order to calculate patient specific probabilities.

Since the data pool for renal patients with serum testing other than PTH and phosphorous was considerable larger (792), we calculated logistic regression coefficients for the variables of age, glucose, hemoglobin, bicarbonate, albumin, creatinine, BUN, potassium, calcium, sodium, alkaline phosphatase, ALT, leukocytes, eGFR and to achieve the most parsimonious model each variable with the least significant value was eliminated in a step wise fashion and the logistic regression recalculated. The final list consisted of age, hemoglobin, albumin, creatinine, ALT, and eGFR.

This calculation had p < 0.005 and a Chi-Square Goodness of Fit test by the Hosmer-Lemeshow method that was not significant at the 0.40 level. In addition, the association between the response variable and the predicted probabilities had 69.9% concordant pairs.

**Figure 3:**
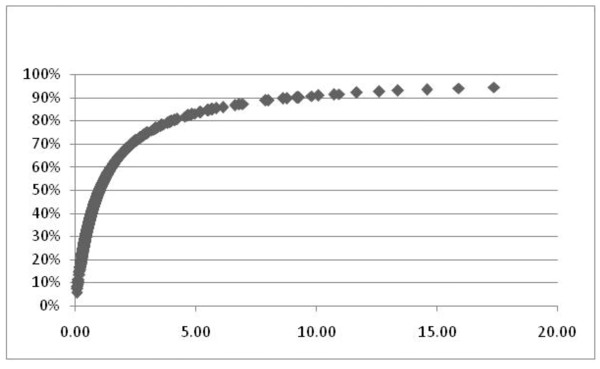
**Probability curve for high cost hospitalization versus the linear regression calculation for age and select blood chemistry values**. Probability for Hospitalization versus the linear progression for age, serum levels for hemoglobin, albumin, creatinine, alanine aminotransaminase, and e-GFR.

Calculation of a probability curve for the outcome of HCH over the study period versus the linear predictor for those variables is displayed in Figure 3.

Figure 3 illustrates the steep rise in probability for HCH to 67% as the linear predictor increased from 0.0 to 2.5. As the curve begins to plateau at a predictor value of 3.0 to 5.0, the probability of HCH increased from 67% to 82%. With an increase in predictor values from 10.0 to 17.0, the probability for HCH rose from 90% to 97%.

**Figure 4:**
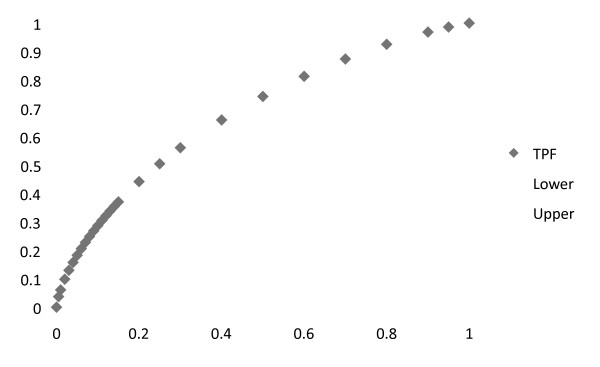
**Receiver operating curve (ROC) curve for PTH, PO4 and serum albumin versus HCH**. The center diamond line represents the true positive fraction(TPF) for HCH in patients with a calculated sum for the linear predictor defined as the linear combination for PTH, PO4 and albumin. The upper and lower gray lines represent the 95% confidence levels. The Y axis = True Positive Fraction (i.e. Sensitivity) versus the X axis for False Positive Fraction (i.e. 1-specificity).

Figure 4 is the ROC area curve based on a sequence of cut-points on the linear predictor defined by the weighted Z scores of PTH, PO4, and albumin.

The Area under the Curve (AUC) shown in Figure 4 was calculated at 0.68. This value was compared to the AUC for a model based on the sum of the non-weighted Z scores for PTH, PO4 and albumin. The AUC for that curve was 0.64. Significance of the difference between these two curves revealed, as expected, a p-value > .05. In a similar manner, the AUC derived from the Z score of PTH alone, as well as for stages of CKD, both had areas of 0.64.

For the cohort of 792 patients, the AUC derived from the linear combination of predictor values for age, serum hemoglobin, albumin, creatinine, ALT and eGFR compared to the true positive occurrence for HCH had an area of 0.699.

**Figure 5:**
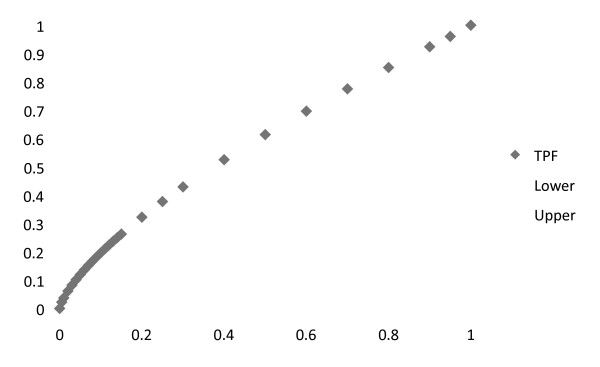
**Receiver operating curve (ROC) for HCH versus CKD stage**. The center diamond line represents the True Positive Fraction (TPF) for HCH compared to the stage of CKD. The upper and lower gray lines represent the 95% confidence levels. The Y axis = True Positive Fraction (i.e. Sensitivity) versus the X axis for False Positive Fraction (i.e. 1-specificity).

In contrast, Figure 5 demonstrates the ROC curve comparing CKD stage to the true positive occurrence of HCH. That AUC was calculated at 0.585. The significance of the difference between the AUC shown in Figures #4 and #5 demonstrated significance at p < 0.005.

In a similar manner, The ROC area curves based on the sum of the non-weighted Z scores for hemoglobin, creatinine, albumin and ALT was calculated at 0.472, and when compared to AUC for Figure #4 demonstrated significance at p < 0.0005. Similarly, the AUC derived from comparison of the average eGFR to the true positive and true negative occurrence of HCH was calculated at 0.414 and when compared to Figure #4 demonstrated a significance at p < 0.0005.

## Discussion

Our study suggests a linear combination of select serum values correlates with prediction of in-patient hospital care (HCH) for CKD patients defined as payments in excess of $3,000 in one or more months over a one year study period.

Although there is controversy in the literature over which mineral metabolites are most significantly related to morbidity and mortality, our investigation found that the sum of a linear combination of beta weighted Z scores for PTH, phosphorous and albumin correlated significantly with the outcome of HCH.

Given the limited pool of 267 patients with regular testing for serum parathyroid hormone and phosphorus, our findings justify further exploration of this promising relationship. Initially we questioned whether patients with tests for PTH and phosphorus had more advanced renal disease than our second cohort of 792 patients without such testing. However the average CKD stage for patients in the first and second cohorts was: 3.8 and 3.6 respectively.

The area under the ROC curve for the linear combination of weighted values for PTH, PO4 and albumin was greater, but not significantly different, than the areas under ROC curves for the non-weighted sum of Z scores for PTH, PO4 and albumin or for the Z score of PTH alone. The association of true positive HCH with the Z score for PTH alone was intriguing to us. The Z scores for average PTH within our patient pool ranged from -2.1 to 79.7, with a mean value of 5.9. This wide variation was not observed in the average Z scores for PO4 or albumin which ranged from -3.6 to 8.1 (mean 0.9), and from -4.9 to 1.2 (mean -1.0) respectively. The wide variation for PTH and its strong correlation with HCH is consistent with other researchers [19]. But this finding is contrasted to other studies analyzing the association of single blood tests to average cost for healthcare [30]. These latter investigators concluded that deviation of single blood tests from their normal range did not predict healthcare costs. We generally agree with that conclusion, and further suggest that measurement of multiple serum variables within a related system such as mineral metabolism disorders or renal dysfunction may improve prediction modeling and correlation to cost. We plan future studies to expand our pool with no missing observations for variables associated with mineral metabolism and renal function. With greater access to data from electronic health records, we postulate that addition of physical measures such as systolic blood pressure and BMI may further improve predictive modeling.

Our second cohort of 792 patients with more complete observations and weighted Z scores displayed better correlation to the true positive occurrence of HCH. That model differed significantly from the model based on non-weighted Z scores of the same blood tests or for stages of CKD.

As public policy supports sizable investments in electronic health records, along with regional health information exchanges, there is rapid movement towards Accountable Care Organizations within the United States. Since ACOs intend to shift provider focus from procedure pricing to better health outcomes, the incentive for achieving this goal is financial compensation based on individual patient outcome. Such a shift will require metrics to predict expected outcomes for patients in various stages of illness. Currently most payers rely on claims data for prediction. Such analysis is population based and does not recognize individual patient complexity.

In order to tailor prevention for better health, improved disease modeling is necessary. Accurate forecasts based on objective data will also enhance delivery of value-based outcomes.

We believe that further investigation is warranted to evaluate additional linear combinations of diagnostic measures for select chronic illnesses in order to achieve these goals.

### Study Limitations

The primary limitations of our study deal with population size, blood test selection by both primary care physicians and specialists, as well as lack of data residing on the medical record such as: micro-albuminuria, systolic and diastolic blood pressure, along with BMI (Body Mass Index). We are engaged in follow on studies, which intend to address these concerns by expansion of our data pool to additional MCO's. With the advent of significant federal and state investments in electronic health records and the establishment of regional health information organizations (RHIO), we have undertaken the necessary consent procedures to acquire more complete physical and laboratory data on confirmed CKD patients.

## Conclusion

In conclusion, our study demonstrates that:

1: A linear combination of blood tests based on Z scores for PTH, PO4, and albumin derived from a multivariate logistic regression model correlates significantly with in-patient hospital payments (HCH) exceeding $3,000 in one or more months over a 13 month study period at p < 0.005.

2: Summing the exponential values for the regression coefficients derived from the logistic regression for those variables divided by one plus the exponential linear progression for those same variables produced a probability curve predicting HCH.

3: Calculation of a probability curve for the occurrence of HCH in one or more months during the study period based on the linear progression of the variables for age, serum hemoglobin, albumin, creatinine, ALT and eGFR demonstrated significance at p < 0.005.

4: Calculation of receiver operating characteristic (ROC) curves for the models predicting HCH based on the linear combination of age, hemoglobin, albumin, creatinine, ALT, and eGFR demonstrated significance at p < 0.005 when compared to ROC area calculations for models based on the non- weighted Z scores for those same variables or CKD stage alone.

5: In contrast, ROC area curves derived from a linear combination of values derived from weighted variables for PTH, PO4, and albumin demonstrated prediction that was better, but not significantly different, than ROC area curves calculated for the non-weighted Z scores for those same variables as well as PTH alone.

6: Our findings suggest that multivariate logistic regression calculations based on blood chemistry values related to illness severity and reimbursement may have value to future accountable care organizations in creating risk adjusted compensation models for providers. In addition, these predictive models may have value in earlier identification of patients for targeted prevention therapy.

## Competing interests

The authors declare that they have no competing interests.

## Authors' contributions

RWB conceived the study design, carried out data analysis, participated in statistical analysis and drafted the manuscript. RLC assisted in data analysis, participated and verified the statistical analysis, participated in drafting the manuscript.

All authors have read and approved the final manuscript.

## Pre-publication history

The pre-publication history for this paper can be accessed here:

http://www.biomedcentral.com/1471-2369/12/65/prepub
